# Gestational Zinc Deficiency Impairs Humoral and Cellular Immune Responses to Hepatitis B Vaccination in Offspring Mice

**DOI:** 10.1371/journal.pone.0073461

**Published:** 2013-09-17

**Authors:** Ning Zhao, Xuelian Wang, Ying Zhang, Qiuhong Gu, Fen Huang, Wei Zheng, Zhiwei Li

**Affiliations:** 1 Department of Infectious Diseases, Shengjing Hospital, Affiliated Hospital of China Medical University, Shenyang, China; 2 The Third Division of Medical Laboratory Technology Center, China Medical University, Shenyang, China; 3 Department of Pathophysiology, China Medical University, Shenyang, China; University of Iowa, United States of America

## Abstract

**Background:**

Gestational zinc deficiency has been confirmed to impair the infant immune function. However, knowledge about effects of maternal mild zinc deficiency during pregnancy on hepatitis B vaccine responsiveness in offspring is limited. In this report, we aimed to examine how maternal zinc deficiency during pregnancy influences humoral and cellular immune responses to hepatitis B vaccination in offspring of BALB/c mice.

**Methodology/Principal Findings:**

From day 1 of pregnancy upon delivery, maternal mice were given a standard diet (30 mg/kg/day zinc), zinc deficient diet (8 mg/kg/day zinc), or combination of zinc deficient diet (8 mg/kg/day zinc in the first 2 weeks of gestation) and zinc supplement diet (150 mg/kg/day zinc for the last week of pregnancy), respectively. Newborn pups of these maternal mice were immunized with hepatitis B vaccine at postnatal weeks 0, 2 and 4. Then, splenocytes and blood samples from the offspring mice were harvested for detection of serum zinc concentrations, humoral and cell-mediated immune responses, expression of cytokines using ELISA, CCK-8 and flow cytometric analysis. Results from the present study demonstrated that gestational zinc deficiency inhibited antibody responses, and decreased the proliferative capacity of T cells in offsprings immunized with hepatitis B vaccine. Additionally, HBsAg-specific cytokines analysis revealed that gestational zinc deficiency could inhibit secretion of IFN-γ from splenocytes, and decrease IFN-γ expression of CD4^+^ and CD8^+^ T cells.

**Conclusions/Significance:**

Gestational zinc deficiency can weaken the humoral and cell-mediated immune responses to hepatitis B vaccine via decreasing B cell counts and hepatitis B virus-specific immunoglobulin G production, as well as reducing T cell proliferation, CD4^+^/CD8^+^ T cell ratio, and Th1-type immune responses.

## Introduction

Hepatitis B virus (HBV) is an important cause of chronic viral infection, with an approximately 400 million chronic carriers in the world [1]. Most carriers of chronic HBV acquire the infection from their mother at birth or within the first one to two years after birth [2]. It is estimated that 14% of female carriers and 50% of male carriers will eventually die of cirrhosis and hepatocellular carcinoma [1]. HBV vaccination is the main strategy for effective control of HBV infection and transmission, which has been recommended by the World Health Organization (WHO) since 1991 [3], [4]. In China, a three-dose series of HBV vaccination with a first dose beginning at birth was introduced in 1992 and generalized in the Expanded Programme of Immunization in 2002 [5]. The HBV vaccination program has successfully decreased the HBV carrier rate in infants from ∼10% to 1% [5], [6], [7] and dramatically reduced the incidence of hepatocellular carcinoma in children [8]. However, ∼4–10% of normal vaccine recipients fail to produce appropriate levels of antibodies after standard immunization [9], [10], [11]. Although the mechanisms underlying the hypo- or nonresponsiveness to HBV vaccine are not fully understood, immunodeficiency has been caused by gestational zinc deprivation [12]. Therefore, the offspring of maternal zinc deficiency during pregnancy might be susceptible to HBV vaccine hypo- or nonresponsiveness.

Zinc deficiency is the 5^th^ leading risk factor for diseases in developing countries and the 11^th^ in developed countries [13]. To date, ∼9.5% of the USA population and ∼33.1% of the Southeast Asia population still suffer from inadequate zinc intake [14]. During pregnancy, zinc requirements are markedly increased and pregnant women are most susceptible to zinc deficiency [15], [16]. In mammals, zinc deficiency is one of the most common causes of immunodeficiency [17], [18]. In addition to the mother’s immune function, gestational zinc deficiency can compromise the infant immunity [12], [19]. Interestingly, a recent study has shown that the efficacy of HBV vaccine is reduced in rats fed with zinc deficient diets [20]. However, whether zinc deficiency during pregnancy leads to HBV vaccine nonresponsiveness is still unknown. In the present study, we focused on the humoral and cellular responses to HBV vaccination of the offspring of mice moderately zinc deprived. We found that the offspring whose mothers had gestational zinc deficiency appeared to a markedly lower cellular and humoral immune response to HBV vaccine, characterized by lower IgG level, weaker T cell proliferation response, and lower expression of IFN-γ by CD4^+^ and CD8^+^ T cells.

## Materials and Methods

### Ethics Statement

All animal experiments were carried out in strict accordance with the animal protection regulations in China and with approval from the Institutional Animal Care and Use Committees of China Medical University (IACUC-L2010567). All efforts were made to minimize animal suffering and the number of animals used.

### Mice and Diet

Fifteen pregnant young adult BALB/c mice, weighing 20.56±2.12 g, were provided by the Center of Experimental Animal, China Medical University (Shenyang, China). The animals were kept in stainless steel housing with wire mesh flooring within a barrier system (22–25°C, 40–60% relative humidity, 12-hour light/12-hour dark cycles), and given ad libitum access to deionized water.

Following pregnancy confirmation, nine maternal mice were randomly given the assigned diets (Trophic Animal Feed High-tech Co., Ltd., China; n = 3 mice for each diet) during pregnancy: 1. Maternal mice were given AIN-93 recommendation diet for rodent (30 mg/kg/d) containing approximately 0.15 mg Zn, and their pups served as controls (n = 16); 2. Maternal mice were given zinc deficient diet (8 mg/kg/d) containing 0.04 mg Zn, and their pups were considered as zinc deficiency group (n = 15); 3. Maternal mice were given zinc deficient diet in the first 2 weeks of gestation and then fed with zinc supplemented diet (150 mg/kg/d) containing 0.75 mg Zn for the last week of pregnancy, and their pups were chosen as zinc supplemented group (n = 17). Another 6 pregnant mice were fed with a normal diet as “wet nurses” for all newborn pups. The diets for all the mice contained a constant protein, caloric, and mineral content (other than Zn).

### Immunization

Newborn pups were immunized intramuscularly via the tibialis anterior muscles beginning at 12 h of birth, and received the secondary intramuscular immunizations with 50 µl/mouse recombinant HBV vaccine (Hansenula Polymorpha) (20 µg/ml, Dalian Hissen Bio-pharm. Co., Ltd., China) at 2 and 4 weeks of age. The pups were weighed at postnatal weeks 0, 2, 4 and 6 and the body weight gain was calculated. Pups were sacrificed at day 14 after the third immunization, and the thymus and spleen were isolated for further analyses. Blood samples from the pups and their mothers were collected and stored at −80°C in trace element-free vials until tested.

### ICP-MS Determination of Zinc Concentrations

All blanks, calibrators, quality control materials and whole blood samples were prepared using diluents containing 0.7 mM NH_4_OH, 0.01 mM Na_2_EDTA*2H_2_O, 0.07% (v/v) Triton-X-100, and 20.00 µg/L internal standard (^72^Ge). The whole blood sample was diluted 50-fold, so that the analyte concentration would fall within the linear dynamic range. Serum zinc concentrations measurements were performed on an Agilent 7700× Inductively Coupled Plasma Mass Spectrometry (ICP-MS, Agilent Technologies, USA). He gas was introduced into the reaction cells.

### ELISA Measurements of HBsAg-specific Antibody

Mouse HBsAg-specific immunoglobulin G (IgG) and IgG isotypes (IgG1 and IgG2a) were quantitatively detected using enzyme-linked immunosorbent assay (ELISA) in accordance with the manufacturer’s instructions. Briefly, 3-fold serial dilutions of sera (100 µl) were added to 96-well microtiter plates that were coated with 0.5 µg/ml HBsAg and blocked with 5% fetal bovine serum in PBS before use. After incubation for 2 h at 37°C, the plates were then washed five times with PBS containing 0.05% Tween 20, and bound antibodies were detected using HRP-conjugated goat anti-mouse IgG, goat anti-mouse IgG1, or goat anti-mouse IgG2a (1∶2000; Chemicon International, USA). Following five washes with PBS, 2,2′-azino-di(3-ethylbenzthiazoline-6-sulfonic acid) (Sigma-Aldrich, USA) was added for dyeing, and the absorbance at 405 nm wavelength was measured using a microplate reader (Sunrise RC, Tecan Group, Maennedorf, Switzerland). Concentrations of anti-HBs antibodies in serum were estimated on a standard curve drawn according to a standard serum mixture from three mice given i.p. injections of 2 µg recombinant HBsAg [11]. The data were expressed as U/ml (1 U = 50% maximum OD).

### Cell Preparation and Culture

The thymus and spleen of pups were removed and weighted. Single cell suspensions from the spleen (referred to as splenocytes hereafter) were prepared by passing through nylon mesh (100 µm pore size, BD Biosciences) with a sterile rubber spatula. Then splenocytes were collected and immersed with lysis buffer (Tiangen Biothch Co., Ltd., Beijing, China) to eliminate red cell. After further washes with PBS×2, the solution were re-suspended in complete RPMI 1640 (Gibco, America) supplemented with 12 mM HEPES (pH 7.1), 0.05 mM 2-mercaptoethanol, 10% (v/v) heat-inactivated fetal bovine serum (FBS, Gibco) and antibiotics (100 IU/ml penicillin, 100 µg/ml streptomycin). The cells were cultured at 37°C in a humidified atmosphere containing 5% CO_2_. Cell numbers were determined with a hemocytometer, and cell viability exceeded 95%.

### Lymphocytes Proliferation Response

Splenocytes were adjusted to a final concentration of 5×10^4^ cells/ml in complete RPMI 1640. The cell suspensions were added to 96-well plates (100 µl/well) in triplicate, and another medium (100 µl/well) containing 2.5 µg/ml concanavalin A (ConA, Sigma, USA), 30 µg/ml bovine serum albumin (BSA, Sigma, USA) or 20 µg/ml recombinant HBsAg was added. After incubated for 72 h, 10 µl cell counting kit-8 (CCK-8; Dojindo Molecular Techinologies, Inc, Japan) was added to each well and the plates were incubated for an additional 1–4 h at 37°C. The absorbance at 450 nm of each aliquot was determined using the microplate reader. The stimulation index (SI) was calculated as the mean reading of the stimulated wells divided by the mean reading of the control wells.

### Flow Cytometric Analysis

Phenotypic analysis of subpopulation of mature T lymphocytes and B lymphocytes from the spleen (2×10^6^ cells/ml) were conducted by using a fluorescent cell counting (FACS Calibur flow cytometer, BD Biosciences, USA) using anti-CD3-PerCP in combination with anti-CD4-FITC or anti-CD8-FITC for T cells and anti-CD19-PE for B cells. Data acquisition and analysis were done with CellQuest software package (version 3.0, BD Biosciences). All samples were analyzed by setting appropriate forward and side-scatter gates around the lymphocytes and the percentage of positive cells was estimated.

### Enumeration of IFNγ- and IL4-secreting Splenocytes

Splenocytes (4×10^6^ cell/ml) suspending in RPMI 1640 containing 10% FBS were added to 96-well plates (100 µl/well) and stimulated with 20 µg/ml recombinant HBsAg (100 µl/well) in triplicate for 72 h. The supernatants of the cultured cells were collected and then loaded onto 96-well plates. IFN-γ and IL-4 were detected with ELISA kits according to the manufacturer’s instructions. The absorbance was recorded at 450 nm using a Sunrise RC microplate reader.

### Intracellular Cytokine Staining of IFNγ and IL-4

Single splenocyte suspensions were prepared on day 14 after the third immunization. T cells at 1×10^6^ cell/well were stimulated in a 6-well plate with recombinant HBsAg (20 µg/ml) or not stimulated for 4 h at 37°C. Then, the plate was incubated with brefeldin A (BFA, 1 µg/ml) for another 24 h and the splenocytes were washed with cold PBS. Cells were blocked with Fcγ-Block (BD phamingen, San Diego, USA) in PBS for 10 min and fixed with 4% formaldehyde solution for 30 min. Cells were suspended in 0.5% saponin for permeabilization and then stained with anti-CD3-PerCP and double stained with anti-CD4-FITC and anti-IFN-γ-PE, or anti-CD4-FITC and anti-IL-4-PE, or anti-CD8-FITC and anti-IFN-γ-PE, or anti-CD8-FITC and anti-IL-4-PE for 30 min at 4°C. Splenocytes were resuspended and transferred to Falcon round-bottom tubes for acquisition on a FACS Calibur flow cytometer, and the results were analyzed using the CellQuest software package.

### Statistical Analysis

Data are expressed as means ± S.D. Statistical significance was determined by one-way analysis of variance (ANOVA) followed by least significant difference post hoc test or Tamhane’s T2 test when appropriate. Body weight was analyzed at different weeks after birth by repeated-measures ANOVA. The significance level of these data was set at *P*<0.05.

## Results

### Thymus and Spleen Weight and Serum Zinc Levels

Two weeks after the beginning of the experiment, zinc-deficient pregnant mice developed mild anorexia and rough hairs. However, the body weight of maternal mice had no difference among the three groups (data not shown). In addition, there were no differences in the body weight (data not shown) and the ratio of thymus or spleen to body weight of the pups in all the experimental groups (*P*>0.05) (Table 1).

**Table 1:** Ratio of thymus or spleen to body weight in the offspring mice whose mothers received different assigned diets during pregnancy.

Variable	Control	ZD	ZS	*P*
	(n = 16)	(n = 15)	(n = 17)	
Thymus weight/body weight,*mg/g*	1.62±0.18	1.61±0.21	1.64±0.15	0.76
Spleen weight/body weight,*mg/g*	3.34±0.13	3.26±0.12	3.28±0.20	0.51

The weight of thymus and spleen were recorded after the offspring were sacrificed. ZD: pups from the zinc deficiency group; ZS: pups from the zinc supplemented group.

**Figure 1:**
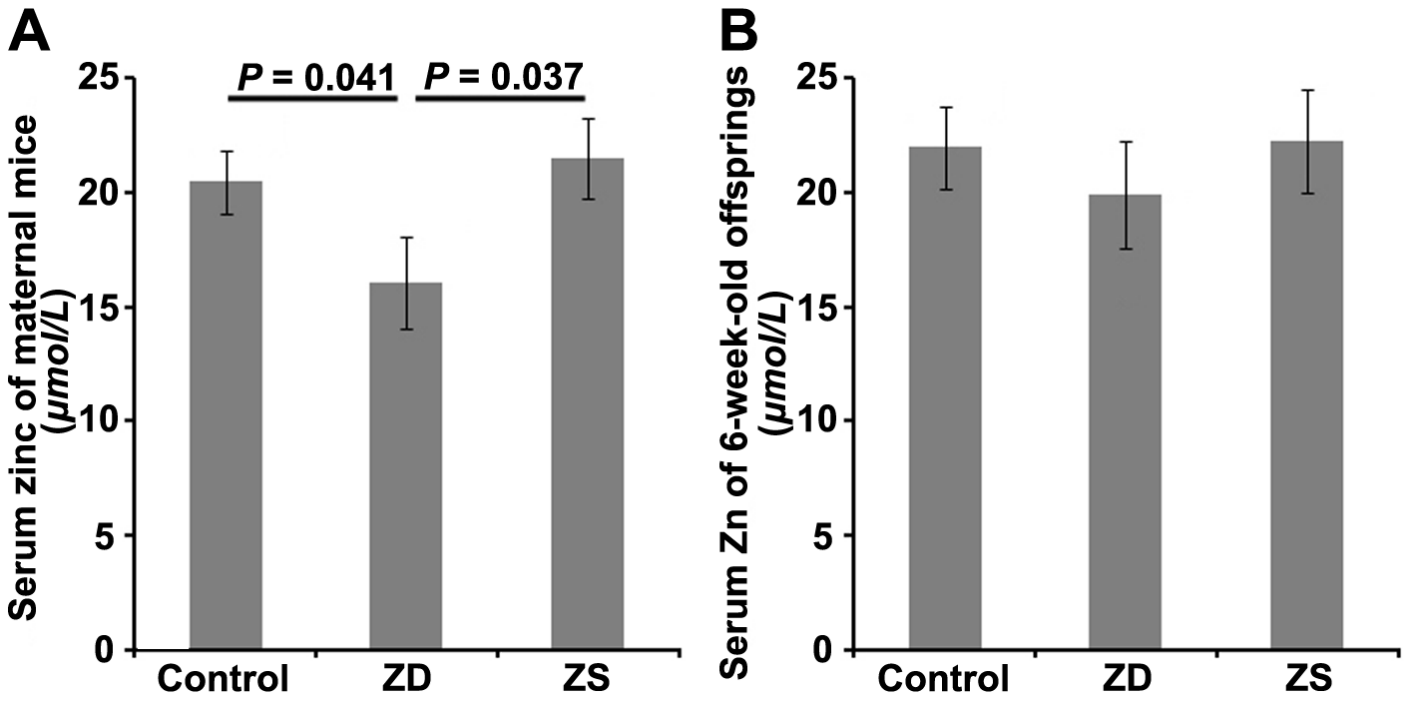
Serum zinc concentration (µmol/L) of maternal mice given different assigned diets during pregnancy (A) and their 6-week-old offspring (B). Serum zinc concentrations measurements were carried out on an Agilent 7700x ICP-MS. The data are expressed as mean ± S.D. ZD: pups from the zinc deficiency group; ZS: pups from the zinc supplemented group.

As the newborn mice were too small to obtain blood samples alive, maternal serum zinc levels were detected to reflect the zinc levels of newborns. Zinc deficiency during pregnancy exerted a significant effect on the maternal serum zinc level. The maternal mice from the zinc deficiency group showed a lowest serum zinc concentration (16.09±2.03 µmol/L) (*P* = 0.041), whereas the serum zinc concentrations of maternal mice from the zinc supplemented group exhibited no significant difference from those of the normal control group. Thus, zinc supplementated mice during late pregnancy showed a better serum zinc level (*P* = 0.037) (Fig. 1A). However, on day 14 after the third immunization, no significant difference was detected in serum zinc levels of the pups among the three groups (Fig. 1B).

### Antigen-specific Humoral Immunity

**Figure 2:**
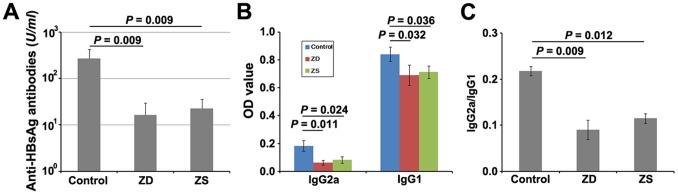
Effect of gestational zinc deficiency on antibody response. (A) Gestational zinc deficiency decreases the titers of HBsAg-specific IgG in serum. (B) Decrease of HBsAg-specific IgG1 and IgG2a in zinc deficiency offsprings. (C) Prenatal zinc deficiency lessens the IgG2a/IgG1 ratio. For all experiments, serum samples from BALB/c mice fed with different zinc content diets were collected on day 14 after the third HBV vaccination and the concentration of anti-HBs IgG, IgG1 or IgG2a was determined by ELISA using a standard curve generated from serially diluted control anti-HBs antibodies. The data are expressed as mean ± S.D. of 5 animals from one representative experiment. Each experiment was performed 3 times. ZD: pups from the zinc deficiency group; ZS: pups from the zinc supplemented group.

To examine the influence of gestational zinc deficiency on humoral responses of offspring mice, the HBV vaccine was inoculated intramuscularly into pups 3 times. On day 14 after the third immunization, serum total IgG, IgG2a and IgG1 against HBsAg were determined by quantitative ELISA. As shown in Fig. 2A, the lowest level of total IgG was detected in the pups from the zinc deficiency group (16.22±13.62 U/ml). Importantly, significant decreases were visible in HBsAg-specific serum IgG2a and IgG1 of the pups from the zinc deficiency group (Fig. 2B). Moreover, the lowest ratio of IgG2a/IgG1 in the zinc deficiency group was induced (Fig. 2C), suggesting impaired Th1 polarization. However, zinc supplementation in late pregnancy appeared to have not enough effect to improve humoral-mediated immune responses to HBsAg.

### T Cell Proliferation and Immune Cell Activation

**Figure 3:**
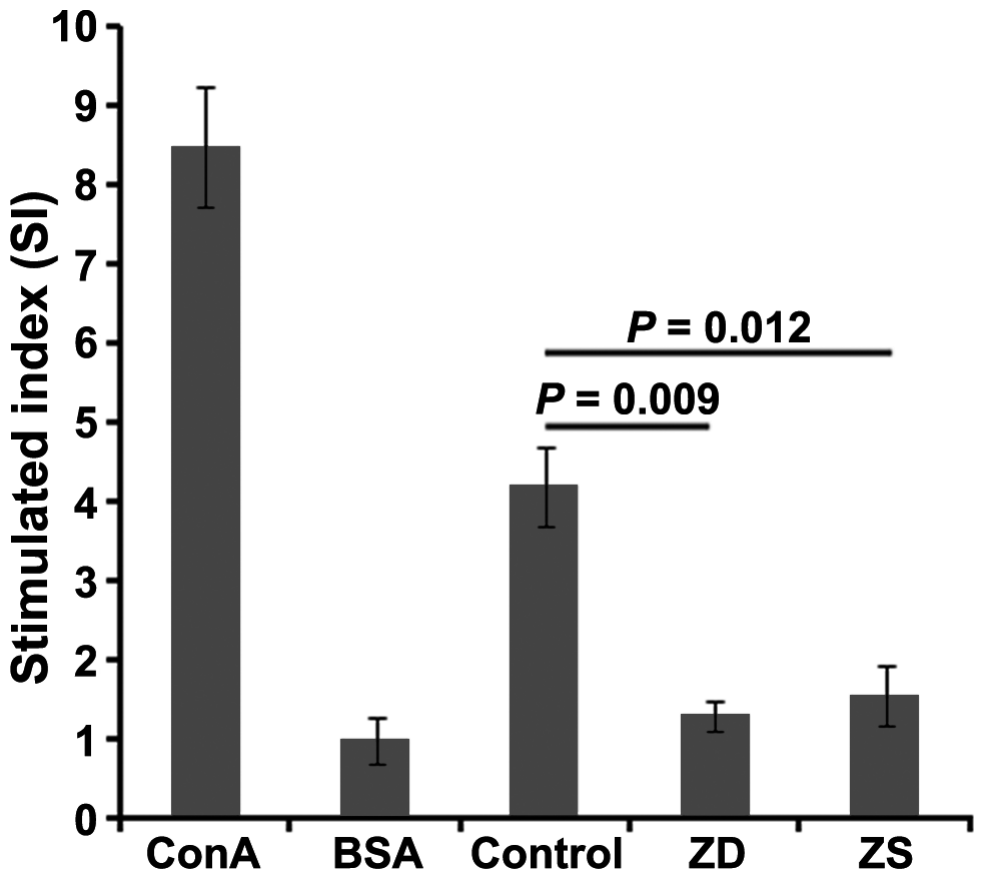
Effect of gestational zinc deficiency on T cell proliferation. Splenocytes from each group were obtained on day 14 after the third HBV vaccination and analyzed for the HBsAg-specific T cell proliferative response, as described in Materials and Methods and expressed as SI. The final concentration in the 96-well plate for ConA, BSA and recombinant HBsAg was 2.5, 30 and 20 µg/ml, respectively. The data are expressed as mean ± S.D. of 3 animals in the proliferative assay from one representative experiment. Each experiment was performed 3 times. ZD: pups from the zinc deficiency group; ZS: pups from the zinc supplemented group.

To further determine whether gestational zinc deficiency influence cell-mediated immunity, a single-cell suspension of splenocytes was re-stimulated with recombinant HBsAg as a specific antigen in culture, and the culture medium served as a negative control. Lymphocytes from a normal pup were re-stimulated in culture with ConA as a positive control, BSA as an irrelevant antigen. As displayed in Fig. 3, the T cell proliferative response to HBsAg of the pups from the zinc deficiency group was significantly decreased compared with the control group, and no significant difference was found between the pups from zinc deficiency group and zinc supplemented group. More importantly, a positive correlation was observed between the SI and IgG level (*r* = 0.64, *P*<0.01).

Total splenocytes were counted after removal of red blood cells and analyzed for T cells (CD3^+^) and B cells (CD19^+^) populations by flow cytometric analysis. As shown in Table 2, there was a remarkable decrease in the total number of splenocytes in the pups from the zinc deficiency group and zinc supplemented group compared to the control group. With regards to T cells and B cells, there was a 2.9-fold and 2.4-fold decrease in T cells and B cells of the zinc deficiency pups compared to the controls, whereas there was no difference in T cells and B cells population between the pups from zinc deficiency and zinc supplemented groups. The above comparison indicated that those cells in the zinc supplemented group pups had no significant increase after zinc supplementation during late pregnancy. Further analysis revealed that gestational zinc deficiency resulted in a change in the percentage of T-cell subpopulations in offsprings after HBV vaccination as compared with normal controls. The numbers of CD4^+^ T cells was lowered in the pups from zinc deficiency and zinc supplemented groups, but the numbers of CD8^+^ T cells showed no difference among groups. A decrease in ratio of CD4^+^ to CD8^+^ was observed in the pups from zinc deficiency group, which was not corrected after supplementation with zinc during late pregnancy (Table 3).

**Table 2:** Immune cell populations in the spleen of offspring mice whose mothers received different assigned diets.

Groups	Total splenocytes(×10^7^)	T cells (×10^7^)	B cells (×10^7^)
Control (n = 16)	16.35±1.32	5.22±1.02	9.76±0.36
ZD (n = 15)	7.61±1.26*	3.55±0.64*	3.58±0.19*
ZS (n = 17)	8.24±2.27*	3.65±0.98*	4.03±0.24*

Total splenocytes were counted after removal of red blood cells and stained for T cells (CD3^+^) and B cells (CD19^+^) on day 14 after the third HBV vaccination. Cells were analyzed by flow cytometric analysis. The number of T cells and B cells in spleen of the pups was counted. ZD: pups from the zinc deficiency group; ZS: pups from the zinc supplemented group.

* *P*<0.05 compared with control.

**Table 3:** CD3^+^ T cell population and its CD4^+^ and CD8^+^ T subpopulations.

Groups	CD3^+^		CD4^+^		CD8^+^		CD4/CD8
	(×10^7^)	(%)	(×10^7^)	(%)	(×10^7^)	(%)	
Control (n = 16)	5.22±1.02	34.70±3.15	2.95±0.58	56.47±4.66	2.17±0.66	41.53±1.99	1.36±0.16
ZD (n = 15)	3.55±0.64*	45.47±1.67*	1.29±0.22*	36.47±3.24*	2.15±0.51*	60.53±1.63*	0.63±0.12*
ZS (n = 17)	3.65±0.98*	40.62±5.24*	1.41±0.39*	38.33±3.73*	2.18±0.79*	59.77±2.18*	0.61±0.14*

Total splenocytes were counted after removal of red blood cells and stained for T cell population (CD3^+^), CD4^+^ T cells, and CD8^+^ T cells on day 14 after the third HBV vaccination. Cells were analyzed by flow cytometric analysis. The data above show the numbers and percentages of T cells. ZD: pups from the zinc deficiency group; ZS: pups from the zinc supplemented group.

* *P*<0.05 compared with control.

### HBsAg-specific INF-γ and IL-4 Expressions and Secretion in T Cells

**Figure 4:**
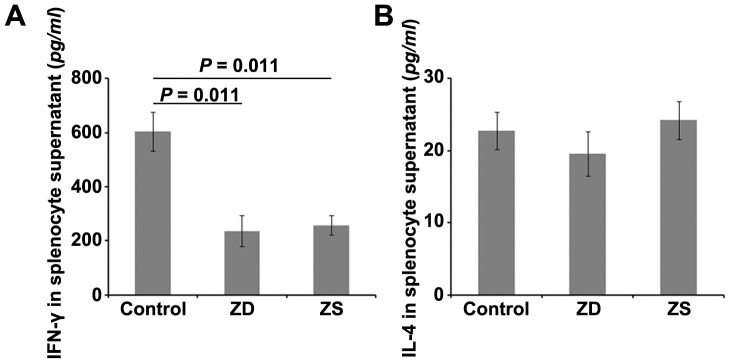
Effect of gestational zinc deficiency on the secretion of cytokines. Splenocytes isolated from the spleen of offspring BALB/c mice on day 14 after the third HBV vaccination were stimulated with recombinant HBsAg for 72 h. Culture supernatants were harvested, and the production of IFN-γ (A) and IL-4 (B) was detected by ELISA. The data are expressed as mean ± S.D. for 5 animals in the enumeration of cytokines-secreting from one representative experiment. ZD: pups from the zinc deficiency group; ZS: pups from the zinc supplemented group.

To evaluate the secretion of cytokines in the CD4^+^ T cells and CD8^+^ T cells, the splenocytes were isolated on day 14 after the third immunization and re-stimulated in culture with the recombinant HBsAg. We measured the secretion of IFN-γ (Th1 like) and IL-4 (Th2 like) from the culture supernatants upon re-stimulation with recombinant HBsAg. As shown in Fig. 4, splenocytes of the pups from zinc deficiency and zinc supplemented groups produced lower levels of HBsAg-specific IFN-γ than the control group, whereas no remarkable changes in production of IL-4 in the three groups.

**Figure 5:**
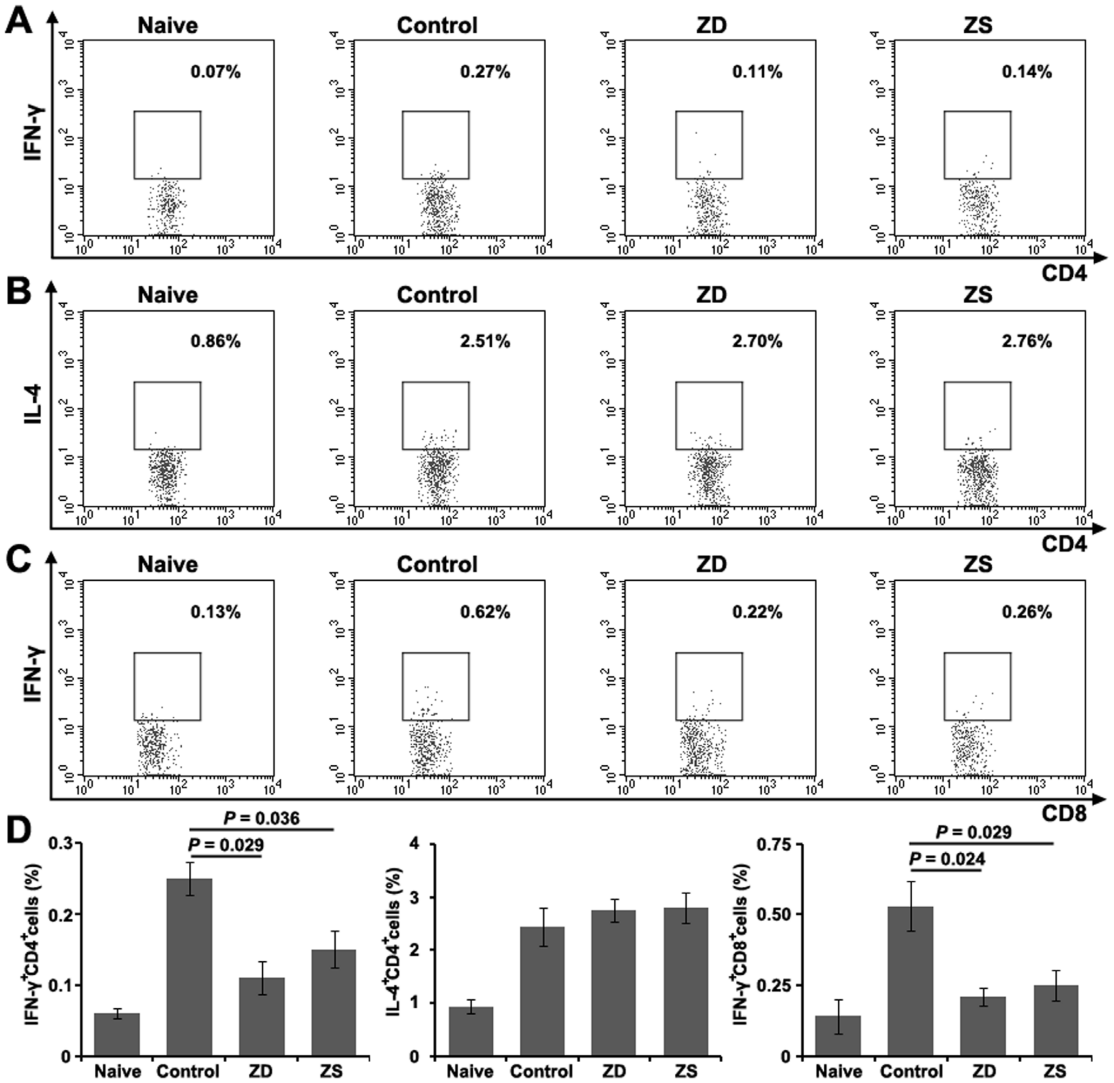
Effect of gestational zinc deficiency on HBsAg-specific cytokine expressions in T cell subsets. Splenocytes isolated from the spleen of offspring BALB/c mice on day 14 after the third HBV vaccination were re-stimulated with recombinant HBsAg. Intracellular staining for IFN-γ, IL-4 in CD4^+^ T cells and IFN-γ in CD8^+^ T cells was performed. The summaries of percentage of IFN-γ in total CD4^+^ (A), IL-4 in total CD4^+^ (B) and IFN-γ in total CD8^+^ T cells (C) are shown (D). The data are expressed as mean ± S.D. of 5 animals in the intracellular cytokine staining assay from one representative experiment. ZD: pups from the zinc deficiency group; ZS: pups from the zinc supplemented group.

To evaluate the expression of cytokines in the subsets of T cells, splenocytes suspension was prepared on day 14 after the third immunization and re-stimulated with recombinant HBsAg. These cells were then analyzed by FACS with a gate set on CD4^+^ and CD8^+^ T cells. From the results shown in Fig. 5A–C and a sum of 5 independent experiments in Fig. 5D, we observed the lowest level of IFN-γ expression for the HBsAg-specific CD4^+^ and CD8^+^ T cells in the pups from zinc deficiency group, whereas the percentage of IL-4 in CD4^+^ T cells was not changed compared with the control group. There was no difference in cytokine expressions between the pups from zinc deficiency group and the pups from zinc supplemented group. This result suggests that gestational zinc deficiency can inhibit the response of both HBsAg-specific Th1 and CD8^+^ effector cells.

## Discussion

In the present study, we demonstrated that newborn pups whose mothers suffered from gestational zinc deficiency failed to develop an adequate IgG level and T cell proliferation response to HBV vaccine. Gestational zinc deficiency due to imbalances between intake and increased requirements is a common world-wide problem. Maternal zinc deficiency can impaire lymphocyte mitogenic responses and depress immunoglobulin concentrations of the offspring [21]. Interestingly, the adverse effect on the infant immune system might even be permanent, persisting after restoration of normal zinc intake [12], [19]. Consistent with these findings, in this experiment, no significant differences were detected in serum zinc levels of the pups at 6 weeks of age among the three groups, whereas both humoral and cell-mediated immune responses were impaired in the pups from zinc deficiency group and zinc supplemented group. Some immune defects caused by prenatal zinc deficiency, such as hypogammaglobulinaemia, altered B cell antibody repertoires together with decreased antigen-specific T cell proliferation, may lead to an impaired success of vaccination in newborns [22]. Whereas nonresponders to the vaccine present dramatically reduced serum zinc levels, responders exhibit similar zinc levels as age-matched controls [23]. There are many different studies concerning the association between post-vaccination antibody response and zinc deficiency [24], [25], especially study of Ozgenc et al [20]. They first documented that marginal zinc deficiency influenced the efficacy of HBV vaccination in young adult rats via inhibiting humoral and cell-mediated immune responses. Therefore, we proposed that gestational zinc deficiency might be one of the important risk factors for hypo- and nonresponsiveness to HBV vaccine.

Previous studies have shown that unlike other immune cells, B cells are not strictly zinc-restricted cells. The number of antibodies produced per activated B cell remains stable in zinc-deprived mice immunized with a T cell-dependent antigen [26]. However, our data have shown that prenatal zinc deficiency reduces the HBV-specific IgG production. It is most likely correlated with the decrease in the number of B cells, which is in accordance with previous reports that zinc deprivation results in impaired lymphopoiesis and pre-B cell development due to higher levels of apoptosis [27], while B cells depend on zinc for proliferation [28], thereby leading to a decrease of the antibody-mediated response.

Moreover, the HBsAg-specific antibody production is T cell-dependent and requires Th cell activation. In the present study, we observed a significant decrease in the number of CD4^+^ T cells, CD4^+^/CD8^+^ ratio and a poor T cells proliferative response to HBsAg in vitro in prenatal zinc deficiency mice. What’s more, a positive correlation was observed between the SI and IgG level, indicating that both quantity and function of T cells are associated with immunological effect of HBV vaccine. Previous studies have shown that good antibody response to HBV vaccine is associated with higher CD4^+^ T cell counts [29], [30] or high CD4^+^/CD8^+^ ratio [31]. Moreover, zinc is required for the regeneration of CD4^+^ T cells [32], [33], and zinc deficiency is associated with significantly decreased CD4^+^/CD8^+^ ratio and lowered B cell numbers [34], which is similar to our findings. Our analysis of HBsAg-specific cytokines showed that prenatal zinc deficiency decreased the expression and secretion of IFN-γ, but not IL-4. The mechanism is possibly due to reduction in T cell counts, as well as inhibition of IFN-γ expression in CD4^+^ T and CD8^+^ T cell induced by prenatal zinc deficiency. IFN-γ is an indispensable part of humoral immune response because it is a key factor involved in IgG production of B cells. As described in the present study, compared to the control pups, prenatal zinc deficiency not only inhibited production of IgG1 and IgG2a, but also decreased the IgG2a/IgG1 ratio. The Th1 response is characterized by an enhancement of IgG2a and the production of IFN-γ. The Th2 response is characterized by an enhancement of IgG1 and the production of IL-4. Therefore, these results indicate that gestational zinc deficiency can suppress Th1-type immune responses. Since HBsAg-specific IgG secretion after HBV vaccination is generated via B-cell activation by CD4^+^ Th1-helper responses [35], [36], we speculate that the reduction in CD4^+^ T counts and functional defects induced by gestational zinc deficiency could weaken B cells activation and proliferation, and eventually result in a decrease in HBsAg-specific IgG secretion.

Numerous vaccination studies with additional zinc supplementation have not revealed an increased antibody titer against the vaccine [37], [38], [39]. In the present study, we showed that zinc supplementation at the end of pregnancy has only a weaker effect on B cell numbers and humoral immune response in the neonates after HBV vaccination. One reason is that, in the majority of these studies, zinc may have been applied in excessively high concentrations, thereby suppressing Th immunity which is required for B cell-induced antibody production. The other reason is that the duration of zinc administration prior to vaccination is insufficient. As B cell activation mainly depends on Th cell stimulation, restoring adequate zinc levels for proper Th cell function needs to be achieved. Correspondingly, Favier and colleagues showed that mothers’ serum zinc level increased after supplementation during the first trimester [40]. Duchateau et al. started zinc supplementation one month prior to vaccination and the IgG response was indeed improved [41]. A vaccination study addressing oral administration of inactivated cholera vaccine in young children in a cholera endemic country revealed an enhancing effect of zinc supplementation every day for 42 days (starting 3 weeks prior to vaccination) on the Th1 response, IFN-γ secretion and the vibriocidal antibody response, particularly in formerly zinc-deficient children [42]. These results indicate that zinc supplementation may benefit the immune response to vaccination in zinc deficient individuals. Nevertheless, the duration and doses of zinc supplementation should be considered carefully because zinc overload might inhibit T cell function [43], [44] or induce copper deficiency anaemia [45], and consequently, result in reduced B cell antibody production [46]. Therefore, this may require a longer period of low-dose zinc supplementation in mothers during pregnancy, rather than provided only in the late of pregnancy before immunization with HBV vaccine.

In summary, the data presented in our study revealed that gestational zinc deficiency could weaken the humoral and cell-mediated immune responses to HBV vaccine. Decreased B cell numbers and HBV-specific IgG production, together with reduced T cell proliferation, CD4^+^/CD8^+^ T cell ratio, and Th1-type immune response were observed in newborn mice whose mothers suffered from gestational zinc deficiency. However, whether this phenomenon may also occur in human and whether a longer period of low-dose zinc supplementation during pregnancy can reinforce immune responses to HBV vaccination should be further investigated in the future.
